# External chest-wall compression in prolonged COVID-19 ARDS with low-compliance: a physiological study

**DOI:** 10.1186/s13613-022-01008-6

**Published:** 2022-04-12

**Authors:** Luca Bastia, Emanuele Rezoagli, Marcello Guarnieri, Doreen Engelberts, Clarissa Forlini, Francesco Marrazzo, Stefano Spina, Gabriele Bassi, Riccardo Giudici, Martin Post, Giacomo Bellani, Roberto Fumagalli, Laurent J. Brochard, Thomas Langer

**Affiliations:** 1 Neurointensive Care Unit, ASST Grande Ospedale Metropolitano Niguarda, Milan, Italy; 2 School of Medicine and Surgery, University of Milano-Bicocca, Milan, Italy; 3 Department of Emergency and Intensive Care, ASST Monza, San Gerardo Hospital, Monza, Italy; 4 Department of Anesthesia and Critical Care, ASST Grande Ospedale Metropolitano Niguarda, Milan, Italy; 5 Translational Medicine Program, Hospital for Sick Children, Toronto, ON Canada; 6 Interdepartmental Division of Critical Care Medicine, University of Toronto, Keenan Research Centre, Li Ka Shing Knowledge Institute, St Michael’s Hospital, 209 Victoria Street, Room 4-08, Toronto, ON M5B 1T8 Canada

**Keywords:** COVID-19, ARDS, Respiratory mechanics, Mechanical ventilation, Ventilator-induced lung injury, Chest-wall compression, Driving pressure, Gas exchange

## Abstract

**Background:**

External chest-wall compression (ECC) is sometimes used in ARDS patients despite lack of evidence. It is currently unknown whether this practice has any clinical benefit in patients with COVID-19 ARDS (C-ARDS) characterized by a respiratory system compliance (*C*_rs_) < 35 mL/cmH_2_O.

**Objectives:**

To test if an ECC with a 5 L-bag in low-compliance C-ARDS can lead to a reduction in driving pressure (DP) and improve gas exchange, and to understand the underlying mechanisms.

**Methods:**

Eleven patients with low-compliance C-ARDS were enrolled and underwent 4 steps: baseline, ECC for 60 min, ECC discontinuation and PEEP reduction. Respiratory mechanics, gas exchange, hemodynamics and electrical impedance tomography were recorded. Four pigs with acute ARDS were studied with ECC to understand the effect of ECC on pleural pressure gradient using pleural pressure transducers in both non-dependent and dependent lung regions.

**Results:**

Five minutes of ECC reduced DP from baseline 14.2 ± 1.3 to 12.3 ± 1.3 cmH_2_O (*P* < 0.001), explained by an improved lung compliance. Changes in DP by ECC were strongly correlated with changes in DP obtained with PEEP reduction (*R*^2^ = 0.82, *P* < 0.001). The initial benefit of ECC decreased over time (DP = 13.3 ± 1.5 cmH_2_O at 60 min, *P* = 0.03 vs. baseline). Gas exchange and hemodynamics were unaffected by ECC. In four pigs with lung injury, ECC led to a decrease in the pleural pressure gradient at end-inspiration [2.2 (1.1–3) vs. 3.0 (2.2–4.1) cmH_2_O, *P* = 0.035].

**Conclusions:**

In C-ARDS patients with *C*_rs_ < 35 mL/cmH_2_O, ECC acutely reduces DP. ECC does not improve oxygenation but it can be used as a simple tool to detect hyperinflation as it improves *C*_rs_ and reduces *P*_pl_ gradient. ECC benefits seem to partially fade over time. ECC produces similar changes compared to PEEP reduction.

**Supplementary Information:**

The online version contains supplementary material available at 10.1186/s13613-022-01008-6.

## Background

SARS-CoV-2 can lead to severe respiratory failure (C-ARDS) with some clinical and radiological characteristics that match the presentation of acute respiratory distress syndrome (ARDS) [1–3]. The management of mechanical ventilation of C-ARDS does not differ much from classic ARDS, with general aims to maintain adequate gas exchange and prevent ventilator-induced lung injury (VILI) with protective ventilation with low tidal volume (*V*_t_), low driving pressure (DP) and by the use of prone position [4–7]. A subset of patients with C-ARDS suffers from a significant reduction in respiratory system compliance (*C*_rs_); this seems to be especially represented in C-ARDS patients needing prolonged mechanically ventilation due to unresolving respiratory failure [8, 9]. Due to this decrease in *C*_rs_, even low tidal volumes (i.e., below 6 mL/kg) often produce high DP values given the existing relationship between DP and *C*_rs_ (i.e., DP = *V*_t_/*C*_rs_) [4]. Recent reports also mentioned the paradoxical positive effects of different supine body positions as well as chest or abdominal compression on respiratory mechanics in such patients [10–14].

In patients with prolonged C-ARDS and low C_rs_ at higher risk of VILI, we used the application of an external chest-wall compression (ECC) [10–13]. We hypothesized that this could reduce regional hyperinflation and reduce the pleural pressure gradient. We, therefore, decided to conduct a prospective physiological study.

The primary aim of the study was to determine if the application of ECC in patients with prolonged C-ARDS and low *C*_rs_ leads to a decrease of the DP and would indicate the presence of regional hyperinflation. We hypothesized that ECC can reduce ventral hyperinflation improving *C*_rs_ and reduce DP. Secondary aims were (1) to assess the consequences of ECC on ventilation distribution, partitioned respiratory mechanics, shunt fraction and dead space, and (2) to compare the effect of ECC and PEEP reduction. Besides respiratory mechanics static measurements, esophageal pressure (*P*_es_) and electrical impedance tomography (EIT) were analyzed to understand partitioned respiratory mechanics and regional distribution of ventilation during the protocol. Moreover, we performed a preclinical study in a porcine model of acute ARDS in which ECC was applied and pleural pressure (*P*_pl_) catheters were employed to test the possibility that ECC decreases the *P*_pl_ gradient.

## Materials and methods

### Human study

This single center physiological study was approved by the institutional review board (Comitato etico Milano Area 3, # 179-30032021). Informed consent was obtained according to Italian regulations. Patients admitted to the COVID-19 ICU (Rossini) of the ASST Grande Ospedale Metropolitano Niguarda, Milan for C-ARDS were enrolled. The study was performed on a convenience sample of 11 patients.

#### Study protocol

- Inclusion criteria were the following: (1)  > 18 years; (2) diagnosis of C-ARDS (laboratory confirmation of SARS-CoV-2 infection and concomitant ARDS according to Berlin definition [15]); (3) mechanically ventilated (Evita V800, Dräger, Lübeck, Germany) with sedation and myorelaxation in volume-controlled mode; (4) protective *V*_t_ (≤ 6 mL/kg) and (5) *C*_rs_ ≤ 35 ml/cmH_2_O on clinical settings.
- Exclusion criteria were: (1) Pregnancy; (2) hemodynamic instability; and (3) Contra-indication to electrical impedance tomography (EIT) positioning (e.g., trauma, burns, pace-maker, defibrillator).

The study protocol had four steps (Additional file 2: Figure E1): (1) at baseline (before the positioning of a weight on the chest wall), we ensured that hemodynamics was stable; then, during an expiratory hold maneuver, we performed a brief static chest compression with a 5-L saline bag, and recorded the change in *P*_aw_ determined by this compression (Additional file 2: Figure E2). (2) ECC, with a 5-L bag placed in the middle of the thorax using the sternum as a landmark, for 60 min; (3) ECC discontinuation, 10 min without compression; and (4) PEEP reduction from baseline by the same amount of static pressure developed by the saline bag (step 1).

At enrollment, clinical ventilator settings were used. No standardized protocol to set PEEP was available in the ICU; therefore, PEEP was set upon clinician’s decision in a tertiary referral hospital.

During all steps, the patients were placed in supine flat position (0° trunk inclination) to standardize every measurement [16] and the ventilator settings were unchanged (except for PEEP in step 4). No recruitment maneuvers were performed.

Before the protocol was started, a 5 Fr esophageal balloon (Cooper surgical, Trunbull, CT, USA) was positioned in 9 out of 11 patients enrolled in the study to partition respiratory mechanics. The proper position of the esophageal balloon was ensured [17]. Patient hemodynamics was monitored by a central line and invasive arterial pressure.

At the end of each step, and after 5, 30 and 60 min of step 2 (ECC), we performed expiratory and inspiratory holds to obtain static measurements of airway (*P*_aw_) and esophageal pressure (*P*_es_). The distribution of tidal volume between ventral (non-dependent, regions of interest 1 + 2) and dorsal (dependent, regions of interest 3 + 4) lung areas was assessed through the analysis of EIT data (PulmoVista 500, Dräger, Lübeck, Germany) to obtain a regional *V*_t_ as previously described [18, 19]. In addition, end-expiratory lung impedance (EELI, a surrogate of end-expiratory lung volume [20]) was analyzed by EIT [21]. The following variables were calculated:

- Respiratory system Driving pressure or DP = Plateau Pressure (*P*_plat_) – Total PEEP (set PEEP + intrinsic PEEP).
- Respiratory system compliance or *C*_rs_ = *V*_t_/DP.
- Regional *C*_rs_ = Regional *V*_t_ derived from EIT/DP.
- Transpulmonary pressure (absolute value) or *P*_L_ = *P*_aw_ – *P*_es_.
- Lung compliance or *C*_lung_ = *V*_t_/(*P*_L_ inspiration – *P*_L_ expiration).
- Chest-wall compliance or *C*_cw_ = *V*_t_/(*P*_es_ inspiration – *P*_es_ expiration).

*P*_aw_ and *P*_es_ waveforms as well as EIT data were prospectively recorded and stored for offline analysis.

Arterial and central venous blood samples were obtained to assess gas exchange and to calculate shunt fraction at baseline, at the end of step 2 (60 min of ECC), at step 3 and step 4. Shunt fraction was calculated as follows: (C_c_O_2_ – C_a_O_2_)/(C_c_O_2_ – C_v_O_2_), where C_c_O_2_ represents the O_2_ content of capillary blood, C_a_O_2_ the arterial O_2_ content and C_v_O_2_ the O_2_ content of venous blood. Alveolar dead space was calculated as follows: (P_a_CO_2_ – P_Et_CO_2_)/P_a_CO_2,_ where P_a_CO_2_ is the arterial partial pressure of carbon dioxide (CO_2_) and P_Et_CO_2_ represents the end-tidal CO_2_ value.

Our primary endpoint was the DP change after the application of an ECC. We hypothesized that ECC would produce a decrease in DP.

Secondary endpoints were: change in chest-wall compliance (*C*_cw_); change in lung compliance (*C*_lung_); change in regional *C*_rs_ (i.e., of non-dependent and dependent lung); change in gas exchange, shunt fraction and dead space after ECC.

### Animal study

We performed an experimental porcine study using ECC in a two-hit lung injury model. The aim of the study was to measure directly the effect of ECC on pleural pressure. Ventral and dorsal *P*_pl_ were, therefore, directly measured to understand the effect of ECC on P_pl_ gradient, differentiating between non-dependent and dependent lung areas.

The experiments were conducted in the animal facility of The Hospital for Sick Children Hospital (Toronto, ON, Canada). All experimental procedures followed the guidelines of the Canadian Council on Animal Care and were approved by the Animal Care Committee, Research Institute, The Hospital for Sick Children (protocol number 46420).

#### Animal preparation

Four healthy Yorkshire pigs (32.6 ± 2.1 kg) were sedated and paralyzed. Pigs were intubated and mechanically ventilated in volume-controlled mode in supine position. An esophageal catheter (Nutrivent; Sidam, Mirandola, Italy) was inserted to record P_es_ and positioned as previously described [17, 18]. Pleural pressure (*P*_pl_) was directly recorded in the dorsal and ventral part of the pleural space in the right lung with two balloons (Cooper surgical, Trunbull, CT, USA). To ensure a proper *P*_pl_ measurement, the calibration of pleural catheters was done at each PEEP level and the minimal non-stressed volume of the balloon with a stable *P*_pl_ measure was selected [22].

Afterwards, we established lung injury by a two-hit model: surfactant depletion with saline lavage followed by injurious ventilation, as described previously [18].

### Experimental data and measurement

The mechanical ventilator (GE Carestation 620, Boston, MA, USA) was set as follows: *V*_t_ 6 mL/kg, respiratory rate 25/min, F_i_O_2_ 1.0. Ventral and dorsal pleural pressures were measured during respiratory holds both at end-inspiration and at end-expiration for every PEEP step. Every step lasted 20 min and was done without (first 10 min) and with (second 10 min) ECC using a 2.3 kg sandbag on top of the thorax. The pleural pressure gradient was calculated as follows: *P*_pl_ gradient = *P*_pl_ dorsal–*P*_pl_ ventral and averaged between different PEEP steps.

### Statistical analysis

Data were expressed as mean ± SD or median ± interquartile range, as appropriate. Data were compared using one-way repeated measures ANOVA followed by Newman–Keuls or Sidak–Holm post hoc tests. If both the normality and equal variance tests failed, repeated measures ANOVA on ranks was used. Difference in continuous data in the preclinical model between baseline and ECC was assessed using the Wilcoxon signed-rank test. Statistical analyses were performed using STATA/16 MP (TX, USA), GraphPad Prism 8.0.2 (La Jolla, CA, USA) and Systat software Inc. (Sigmaplot 12.0, UK). Statistical significance was set at *P* < 0.05 (two-tailed).

## Results

### Patients

Among the 11 patients, 7 were male and were studied on average 17.3 ± 8.6 days from mechanical ventilation initiation. *C*_rs_ was 25.9 ± 5.9 mL/cmH_2_O, *V*_t_ was 5.4 ± 0.6 mL/kg of PBW (i.e., 365 ± 72 mL). Patients had a median P_a_O_2_/F_i_O_2_ ratio of 163 mmHg (109–220) and moderate to severe hypercapnia (PaCO_2_ = 55.9 ± 6.6 mmHg) with a ventilatory ratio of 2.1 ± 0.4 [23]. Table 1 summarizes baseline and physiological characteristics of the studied population.

**Table 1:** Baseline demographic and clinical characteristics

Demographic characteristics	
Male, *n* (%)	7 (64%)
Age (years)	59 ± 14
BMI (kg/m^2^)	29 ± 8
Clinical illness severity	
Time from disease onset to ICU admission, days	9 (6–10)
Time from ventilation initiation to enrollment, days	17.3 ± 8.6
Time from disease onset to enrollment, days	26.0 ± 8.2
Chronic APACHE	
A B C D	6 (55%) 5 (45%) 0 0
Comorbidities	
Coronary artery disease, *n* (%)	1 (9%)
Hypertension, *n* (%)	4 (36%)
Diabetes, *n* (%)	2 (18%)
Neoplasia, *n* (%)	3 (27%)
Ventilatory variables at enrollment	
Tidal volume (mL)	364.5 ± 72.0
Tidal volume per predicted body weight (mL/kg of PBW)	5.38 ± 0.58
Respiratory rate (breaths per minute)	24 ± 2
Total PEEP (cmH_2_O)	12.6 ± 2.9
Peak inspiratory pressure (cmH_2_O)	32.2 ± 2.2
Plateau pressure (cmH_2_O)	26.8 ± 2.4
Driving pressure (cmH_2_O)	14.2 ± 1.2
Respiratory system compliance (mL/cmH_2_O)	25.9 ± 5.9
Lung compliance (mL/cmH_2_O)	28.7 ± 6.1
Chest-wall compliance (mL/cmH_2_O)	152 (120.2–305.1)
Mean airway pressure (cmH_2_O)	17.0 ± 2.8
Minute ventilation (L)	8.8 ± 2.3
Ventilatory ratio	2.1 ± 0.4
Inspiratory esophageal pressure (cmH_2_O)	14.9 ± 3.4
Expiratory esophageal pressure (cmH_2_O)	12.8 ± 3.1
Gas exchange at enrollment	
P_a_O_2_ (mmHg)	88.2 (77.1–127.6)
P_a_CO_2_ (mmHg)	55.9 ± 6.6
pH	7.371 ± 0.03
P_a_O_2_/F_i_O_2_ (mmHg)	163 (109–220)
F_i_O_2_ (%)	60 (50–65)
End-tidal CO_2_ (mmHg)	42 (39–50)
Dead space (%)	18.6 (12.6–27.1)
Shunt fraction (%)	15.5 (7.1–27.3)

Data are expressed as *N* (%), median and interquartile range or mean ± standard deviation as appropriate

*BMI* body mass index, *ICU* intensive care unit, *PBW* predicted body weight, *PEEP* positive end-expiratory pressure

### Effect of ECC on driving pressure

As compared to baseline, the ECC caused a significant decrease in DP after 5 min (14.2 ± 1.3 vs*.* 12.3 ± 1.3 cmH_2_O, *P* < 0.001) (Additional file 1: Table E2). After 30 and 60 min from the beginning of the ECC, the DP was still significantly lower as compared to baseline values; however, a slight increase of DP over time was observed (12.7 ± 1.4 cmH_2_O at 30 min and 13.3 ± 1.5 cmH_2_O at 60 min, respectively, *P* < 0.001 and *P* = 0.03 as compared to baseline). Once compression was discontinued the DP returned to baseline values (14.9 ± 1.8 vs. 14.2 ± 1.3 cmH_2_O, *P* = 0.3).

### Effect of PEEP reduction

**Fig. 1:**
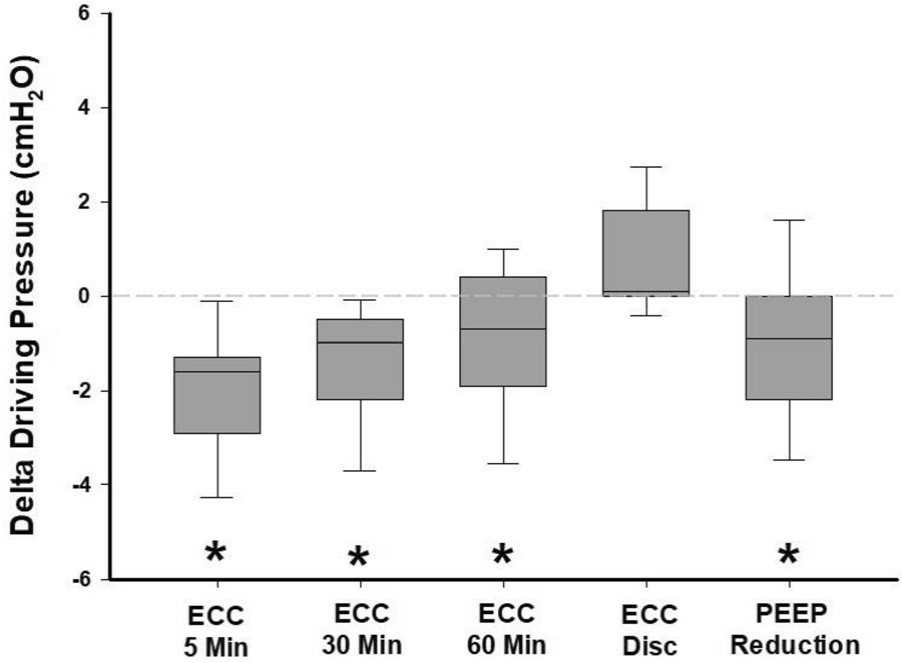
Delta Driving Pressure from Baseline. *N* = 11. Variation in driving pressure (timepoint driving pressure—baseline driving pressure) over the study. Data are represented by box plots and are expressed in cmH_2_O. The horizontal dashed line represents baseline. *ECC* external chest-wall compression, *ECC Disc* ECC discontinuation. *P < 0.05 of absolute values of different timepoints vs. baseline

**Fig. 2:**
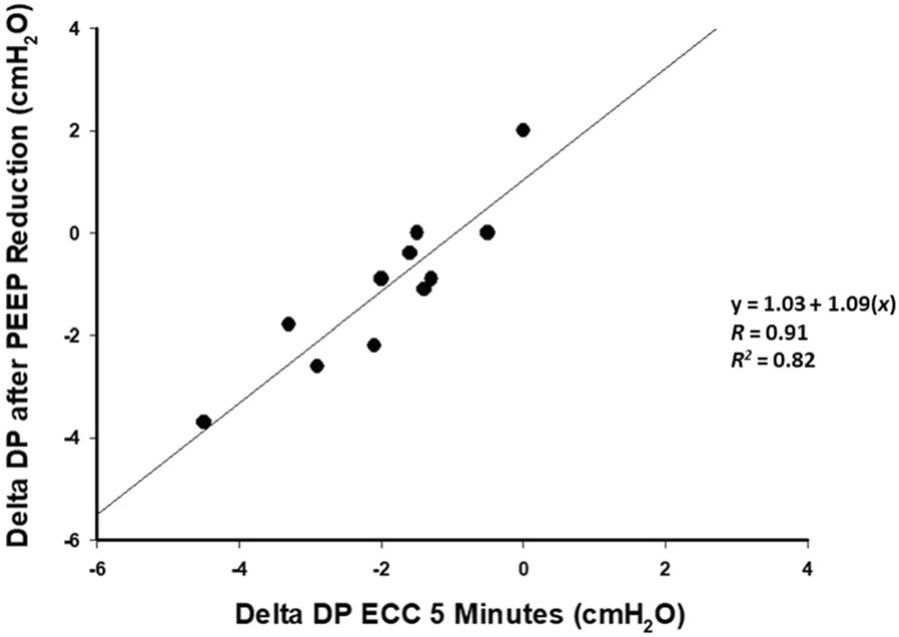
Change in driving pressure produced by ECC vs. change in driving pressure produced by PEEP reduction. *N* = 11. Relationship between variations in driving pressure obtained after 5 min of ECC (5 min ECC driving pressure—baseline driving pressure) and variations in driving pressure obtained after PEEP reduction (PEEP reduction driving pressure—baseline driving pressure). A strong correlation (*R*^2^ = 0.82, *P* < 0.001) was observed between these variables. *ECC* external chest-wall compression, *DP* driving pressure

According to the measurements performed in Step 1, the increase in airway pressure caused by ECC performed during an expiratory hold, PEEP was reduced by 3 cmH_2_O in every patient during the last step (Additional file 2: Figure E2). After the reduction of PEEP by 3 cmH_2_O (i.e., final step) we observed a significant reduction of DP (13.1 ± 1.3 cmH_2_O, *P* = 0.01). The difference of DP between each step and baseline are shown in Fig. 1, absolute DP values are reported in Additional file 1: Table E2. A strong linear correlation was observed between the decrease in DP recorded 5 min after initiation of ECC and the one obtained after PEEP reduction (*R*^2^ = 0.82, *P* < 0.001, Fig. 2; bias = − 0.86 cmH_2_O, upper limit of agreement = 0.40 cmH_2_O, lower limit of agreement = − 2.13 cmH_2_O, Additional file 2: Figure E8).

### Effect of ECC on respiratory mechanics

The ECC caused a significant increase in *C*_rs_ after 5 and 30 min, as compared to baseline values (baseline 25.9 ± 5.9 mL/cmH_2_O, 5 min of ECC 30.2 ± 7.8 mL/cmH_2_O, 30 min of ECC 29.2 ± 7.7 mL/cmH_2_O, *P* < 0.001 for both). After 60 min of ECC and after its discontinuation no significant differences were observed (*P* = 0.06 and *P* = 0.5, respectively). Decreasing PEEP by 3 cmH_2_O produced a significant increase in *C*_rs_ as compared to baseline (28.3 ± 7.8 mL/cmH_2_O, *P* = 0.028, Additional file 1: Table E2).

**Fig. 3:**
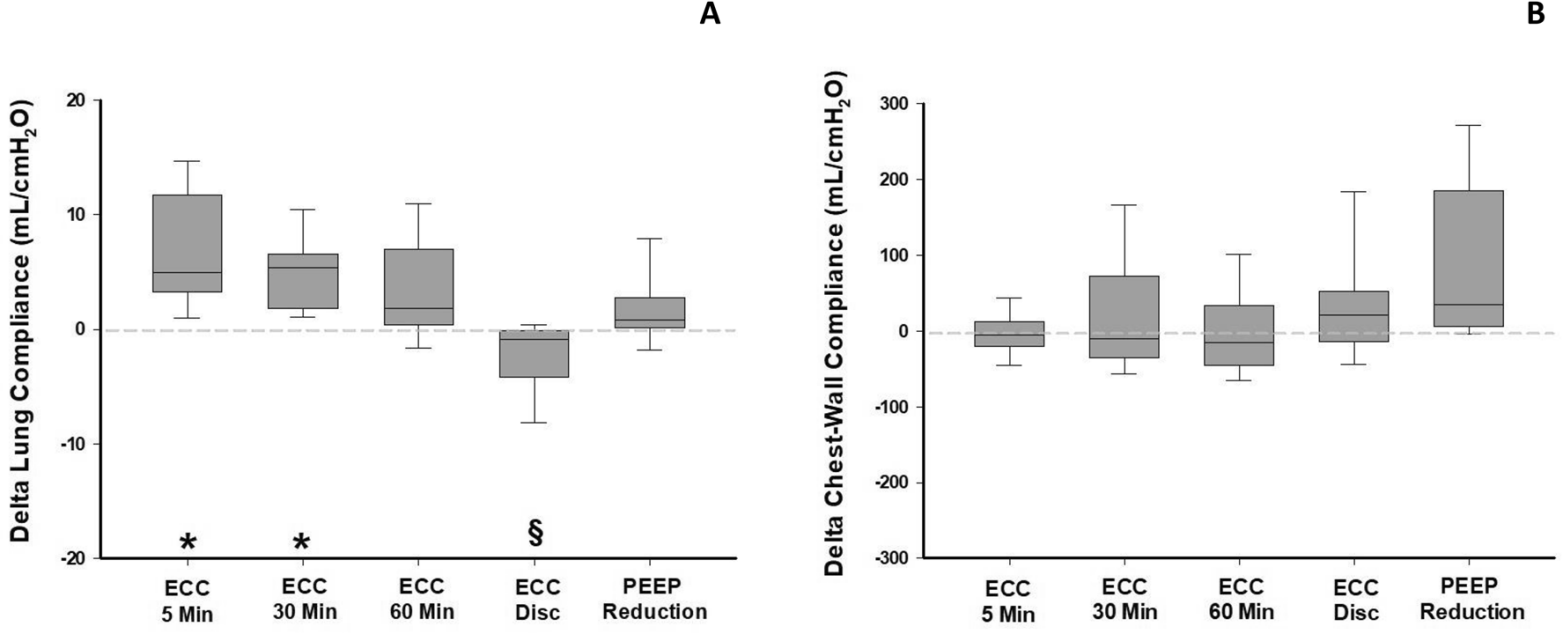
Variations in partitioned respiratory mechanics. *N* = 9. Box plot representation of variations in lung (**A**) and chest-wall (**B**) compliance over the study period. Data are expressed as variation compared to baseline values (timepoint partitioned compliance—baseline partitioned compliance). The horizontal dashed line represents baseline. *ECC* external chest-wall compression. *P < 0.05 of absolute values of different timepoints vs. baseline, §*P* < 0.05 ECC discontinuation vs. ECC at 5, 30 and 60 min

When partitioning respiratory mechanics, we observed that the increase in *C*_rs_ was attributable to an increased *C*_lung_ during the entire ECC period. Compared to baseline values (28.7 ± 6.1 mL/cmH_2_O), *C*_lung_ increased both at 5 (35.5 ± 9.3 mL/cmH_2_O, *P* < 0.001) and 30 min (33.3 ± 8 mL/cmH_2_O, *P* = 0.016) of ECC (Fig. 3A). After 60 min of ECC, *C*_lung_ was still higher as compared to baseline, but the difference was not statistically significant (32.1 ± 7.9 ml/cmH_2_O, *P* = 0.06). In the last two steps (i.e*.*, ECC discontinuation and PEEP decrease) we did not observe difference compared to baseline. When ECC was discontinued *C*_lung_ was significantly lower compared to ECC at 5, 30 and 60 min (26.5 ± 6.3 vs., respectively, 35.5 ± 9.3, 33.3 ± 8.0 and 32.1 ± 7.9 mL/H_2_O). We did not find any significant change of *C*_cw_ over time despite ECC (Fig. 3B).

No statistical difference regarding end-expiratory transpulmonary pressure was found between baseline and each step (Additional file 2: Figure E3, Panel A). On the other hand, the inspiratory transpulmonary pressure (Additional file 2: Figure E3, Panel B) was reduced by ECC, from 11.7 ± 4.3 cmH_2_O (baseline) to 7.7 ± 5.0, 8.1 ± 5.2 and 9.1 ± 5.1 cmH_2_O (respectively, after 5 [*P* < 0.001], 30 [*P* < 0.001] and 60 [*P* = 0.007] min).

### Effect of ECC on gas exchange and hemodynamics

P_a_O_2_ and P_a_O_2_/F_i_O_2_ ratio did not change with ECC. The P_A_O_2_–P_a_O_2_ gradient increased from 254.4 (162.0–327.2) at baseline to 266.4 (164.1–328.7) after 60 min of ECC (*P* = 0.02), (Additional file 1: Table E3). Moreover, there was a trend toward a decrease in CO_2_ with ECC (*P* = 0.06) as underlined by a significant increase in pH (*P* = 0.01). Dead space, shunt fraction and ventilatory ratio were not affected by ECC and PEEP reduction (Additional file 1: Table E3). No statistical difference between steps was found regarding hemodynamic variables (Additional file 1: Table E4).

### Effect of ECC on regional ventilation and compliance

Changes in regional redistribution of ventilation caused by ECC were assessed by EIT and used to derive regional *C*_rs_. Additional file 2: Figure E4 panel A shows the *V*_t_ distribution between non-dependent (ventral) and dependent (dorsal) lung regions across all steps. After 5 min of ECC a slight increase in non-dependent ventilation (62.3 ± 9.2 to 64.7 ± 8.3% (*P* = 0.01)) and a decrease in dependent ventilation (37.7 ± 9.2 to 35.3 ± 8.3% (*P* = 0.01) was observed. No difference was found after 30 and 60 min. After ECC discontinuation, *V*_t_ distribution was similar to baseline (Additional file 2: Figure E4 Panel B). PEEP reduction produced a change in *V*_t_ distribution almost identical to what was observed after 5 min of ECC (non-dependent lung, 64.7 ± 8.3%; dependent lung, 35.3 ± 8.3%, both *P* = 0.01 compared to baseline). A linear association between these two variables was observed (*R*^2^ = 0.51, *P* = 0.01, Additional file 2: Figure E5).

**Fig. 4:**
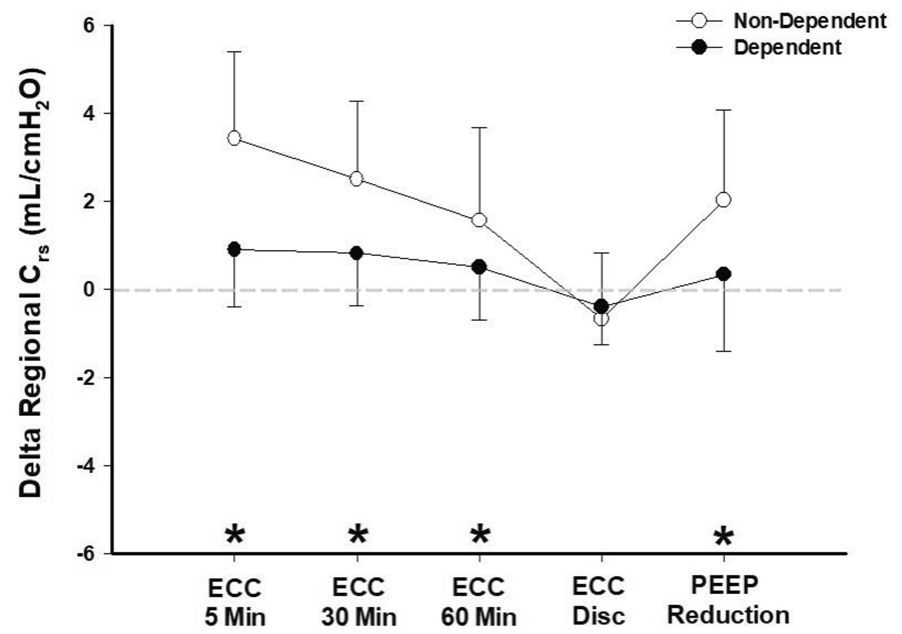
Variations in regional respiratory system compliance (*C*_rs_). *N* = 11. Variation in regional respiratory system compliance (expressed as timepoint regional compliance—baseline regional compliance, *C*_rs_). White dots represent non-dependent lung regions (ventral), black dots represent dependent lung regions (dorsal). *ECC* external chest-wall compression. *P < 0.05 of absolute values of different non-dependent regional *C*_rs_ timepoints vs. non-dependent regional *C*_rs_ baseline

The derived regional *C*_rs_ (i.e., regional *V*_t_/DP) is shown in Fig. 4 and it is expressed as delta regional *C*_rs_ for each step compared to baseline. At baseline, the non-dependent regional *C*_rs_ was 16.4 ± 5.5 mL/cmH_2_O, while it was 9.4 ± 2.2 mL/cmH_2_O for the dependent lung regions. After 5, 30 and 60 min of ECC the non-dependent *C*_rs_ was 19.8 ± 6.6 (*P* < 0.001), 18.9 ± 6.7 (*P* < 0.001) and 17.9 ± 6.5 mL/cmH_2_O (*P* = 0.04), respectively. Finally, when PEEP was decreased the non-dependent regional *C*_rs_ also increased to 18.4 ± 6.1 mL/cmH_2_O (*P* = 0.005). While dependent regional *C*_rs_ did not change significantly with ECC, non-dependent (ventral) *C*_rs_ was always significantly higher during ECC as compared to baseline.

### Effect of ECC on the regional distribution of EELI

Regional EELI values recorded after 5 min of ECC were compared with EELI values obtained at 30 and 60 min, and EELI variations were computed. The analysis was thus performed exclusively during the ECC application to avoid signal distortion caused by the compression of the belt [24]. ECC led to a progressive reduction over time of global EELI (− 277.8 ± 481.7 AU after 60 min ECC, Additional file 2: Figure E6 as an example). Regional analysis showed that the EELI reduction during ECC was in the non-dependent part with an absolute value at 60 min ECC [26.3 (− 55.4–71.8) AU] significantly lower compared to the one observed after 5 min of ECC [415.9 (148.5–639.1) AU, *P* = 0.014] (Additional file 2: Figure E7).

### Animal study: effects of ECC on pleural pressure gradient

**Fig. 5:**
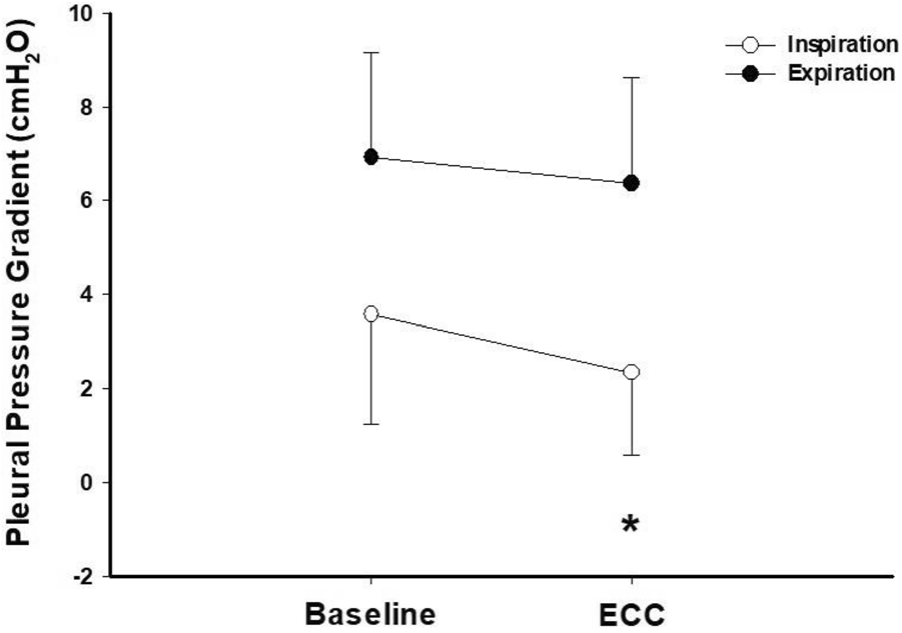
Effect of ECC on pleural pressure (*P*_pl_) Gradient in pigs with experimental ARDS. *N* = 4. *P*_pl_ gradient was calculated as *P*_pl_ dorsal–*P*_pl_ ventral, both at end-inspiration (white dots) and end-expiration (black dots). *ECC* external chest-wall compression. *P < 0.05 vs. baseline

In Fig. 5, we report the effect of ECC on *P*_pl_ gradient on average between different PEEP steps. The *P*_pl_ gradient (i.e., the difference between the dependent and non-dependent pleural pressure), was measured at end-inspiration and was significantly lower with ECC compared to baseline [2.2 (1.1–3.0) vs. 3.0 (2.2–4.1), *P* = 0.035]. On the contrary the *P*_pl_ gradient measured at end-expiration did not change significantly with ECC (*P* = 0.4).

## Discussion

Our study showed that in a population of C-ARDS characterized by a *C*_rs_ below 35 mL/cmH_2_O the application of an ECC led to a rapid decrease of DP and increase in *C*_rs_, explained by an improvement of *C*_lung_. The increase in *C*_lung_ was mainly driven by the non-dependent lung, suggesting a reduction of lung hyperinflation in this lung region. PEEP reduction had comparable mechanical effects. Finally, in an animal model of ARDS, the ECC lowered the *P*_pl_ gradient, suggesting a better parenchymal homogeneity.

### Effect of ECC on respiratory mechanics

After 5 min of an ECC by a 5-L bag we demonstrated that *C*_rs_ increases, leading to a significant decrease in DP. This allowed to improve protective mechanical ventilation by reducing DP for a given *V*_t_. This change in respiratory mechanics could be caused either by a recruitment of previously collapsed alveolar units (usually occurring in the dependent lung) or by a reduction of the overdistended volume, localized predominantly in the non-dependent lung. EIT analysis demonstrated that ECC for 5 min produced a significant increase in non-dependent C_rs_. This finding, along with the rapid reversal of *C*_rs_ once ECC was discontinued, the negative expiratory and inspiratory values of P_L_ and the progressive reduction of end-expiratory lung volume [10, 12], make the hypothesis of dorsal recruitment unlikely and suggest—in contrast—a reduced hyperinflation phenomenon. Indeed, ECC reduced the respiratory system (chest-wall and lung) volume (i.e., EELI) leading to a *C*_lung_ improvement. The mechanism of this regional compliance change is related to a downward shift of the respiratory system pressure (Additional file 2: Figure E13) [12, 13]. Interestingly, *C*_cw_ was not significantly affected by ECC. This is consistent with the effects of obesity, which imposes and extra-load on the chest but does not modify chest wall compliance [25].

The hyperinflation reduction hypothesis was also proposed by Rezoagli et al. [13]. The authors observed, after external chest compression, an EELI reduction and an increase in the non-dependent lung compliance. Other authors reported ECC to be a useful maneuver to attenuate hyperinflation in patients with asthma [26, 27]. On the other hand, a recent case report on C-ARDS supports the dorsal recruitment theory [12]. Given that these findings are based on a single case observation it is difficult to explain the differences with our findings.

Similar studies [13, 14] show that a compression applied to the abdomen either by gravitational forces (Trendelenburg position) or by external compression determines a cephalad displacement of the diaphragm and compresses the lungs, leading to a DP reduction and a better *C*_rs_. Hence, chest-wall and abdominal compressions, although exerting different forces, seem to produce similar effects when applied to a hyperinflated lung.

### Sustained effect of ECC on respiratory mechanics and gas exchange

Lung mechanics after 30 and 60 min showed a progressive loss of the benefit (DP reduction and *C*_rs_ improvement) as compared to the one observed after 5 min of ECC. These data suggest, therefore, a possible time-dependent effect of ECC on respiratory mechanics. Our findings show that ECC plays its role predominantly in the non-dependent lung. At this level, the regional *C*_rs_, after an initial increase, decreases over time. On the contrary, regional *C*_rs_ of the dependent region was fairly stable during the 60 min of ECC. Interestingly, we found a similar behavior of EELI, where ECC seems to selectively determine an EELI loss in the non-dependent lung, resulting in a reduction of the hyperinflated volume. On the other hand, it is conceivable that a long-term ECC could favor the reduction of the non-hyperinflated volume, possibly explaining the observed loss of the benefits to *C*_rs_ over time.

This asymmetrical (ventral vs. dorsal) influence of ECC on respiratory system can be explained also from a pleural pressure perspective, where ECC might cause an asymmetrical change in *P*_pl_ (and *P*_L_) as suggested by our preclinical results (see below).

Despite a study advocating ECC to increase oxygenation [11], we did not observe an increase in P_a_O_2_ or P_a_O_2_/F_i_O_2_ after 60 min of ECC. P_a_CO_2_ was slightly reduced by ECC and can be related to the decrease non-dependent hyperinflation. However, we did not observe any difference in dead space through steps. We acknowledge that these are preliminary physiologic findings and the sample size is not powered to capture significantly differences on gas exchange.

### C-ARDS or ARDS?

The patients enrolled in the study were still on mechanical ventilation despite several days. Hence, they represent a subset of prolonged, unresolving C-ARDS with different mechanical properties compared to early C-ARDS [9]. This population limits generalizability of our data regarding C-ARDS on one hand. On the other hand, our results are likely to be observed in classical ARDS considering the similarities existing between late, low-*C*_rs_ C-ARDS and classical ARDS, as suggested by recent literature [13].

### PEEP titration: is set PEEP too high?

Our data show a robust correlation between the DP reduction after 5 min of ECC and the DP change after PEEP reduction. Therefore, it is possible that short term ECC may inform the clinician about: (1) presence of hyperinflation and (2) the expected DP resulting from a PEEP reduction. The idea of ECC as a bed-side tool has been already proposed, in theory, for abdominal compressions [14, 28]. Our results, strengthen this rationale. Similar findings were reported by Carteaux et al. [12].

Was, therefore, set PEEP too high? Probably yes. Prolonged C-ARDS parenchyma is relatively unmodified by recruitment maneuver and PEEP [9]. In this “fibrosis-like” condition, we observed hyperinflation with slightly negative end-expiratory *P*_L_ (between 0 and − 2 cmH_2_O). Despite recent evidence showed that such *P*_L_ levels were associated with lower mortality [29], no data on outcome are available using this approach in late C-ARDS. Therefore, we might speculate that in this subset of patients PEEP favored more hyperinflation of already aerated lung than recruitment of collapsed alveoli.

### Translational research insight

The porcine experimental data allowed us to measure *P*_pl_ directly in the ventral and dorsal pleural space. Of note, the *P*_pl_ gradient (*P*_pl_ dorsal–*P*_pl_ ventral) is associated to the homogeneity of lung parenchyma assessed with quantitative computed tomography [19, 30, 31]. We found that ECC reduces on average the *P*_pl_ gradient compared to baseline. This reduction implies that ECC increases *P*_pl_ in an asymmetrical way: ventral *P*_pl_, close to the site of ECC, increases more than dorsal *P*_pl_ leading to a greater reduction of *P*_L_ for a given *P*_aw_. Once again, ventral lung seems more affected from the ECC than the dorsal one.

### Clinical implications: ECC as a bedside tool to detect lung overdistension

Based on our data, we think that a brief ECC could be a valuable, quick, and simple bed-side tool to detect hyperinflation, which is a major contributor to VILI. Hence, it can possibly help the clinician adjust PEEP and/or *V*_t_ setting if permitted by the level of pH and P_a_CO_2_. It has been proposed that ECC could be a surrogate of prone position to be used in those patients, where prone position is not feasible [11, 28]. We did not observe an improvement in gas exchange. Moreover, the ECC did not promote recruitment and its effects were time dependent. Indeed, despite a relatively short period of time (1 h) the mechanical benefits of ECC seem to fade over time. This behavior is opposite as compared to prone positioning, where benefits are correlated with time, and therefore, longer durations are encouraged [32].

## Limitations

Our study has some limitations. First, human subjects and pigs differ for amount of weight on the thorax, chest-wall shape and type of lung injury. For this reason, clinical and translational results should be interpreted with caution. Second, this study does not explore ECC with different weights. It is possible that different pressures applied to the respiratory system could yield different mechanical responses. Third, clinical ventilatory settings were used throughout the study as per clinical decision, hence PEEP was not standardized upon a specific titration approach (i.e., incremental vs. decremental PEEP trial, *P*_L_ trial) before enrollment. Fourth, steps were not randomized throughout the study. Fifth, we studied a small convenience sample. Larger studies are, therefore, warranted to confirm our results. Finally, we did not explore the long-term effects of ECC, which will need properly designed studies to be assessed.

## Conclusions

In patients with late C-ARDS, with prolonged mechanical ventilation and low *C*_rs_, ECC can suggest a decrease in hyperinflation in the non-dependent lung regions and leads to a sudden improvement of *C*_rs_, at least transiently. The extent of *C*_rs_ improvement with ECC is similar to the improvement obtained with decreasing PEEP. A brief ECC could, therefore, be a simple and useful tool to detect hyperinflation at the bedside. Over time, the “VILI-sparing” effect of ECC is gradually lost and thus, ECC may not be suitable for a prolonged use (i.e., > 1 h). Hemodynamics and gas exchange are not affected by 1 h of chest compression.

## Supplementary Information

**Additional file 1.** Contains legends for additional figures and additional tables.

**Additional file 2.** Contains additional figures.

## Data Availability

The data sets used and/or analyzed during the current study are available from the corresponding author on reasonable request.

## Abbreviations

ECC: External chest-wall compression
PEEP: Positive end-expiratory pressure
ARDS: Acute respiratory distress syndrome
Sars-CoV-2: Severe acute respiratory system coronavirus 2
C-ARDS: COVID-19 ARDS
VILI: Ventilator-induced lung injury
*V*_t_: Tidal volume
DP: Driving pressure
*C*_rs_: Respiratory system compliance
EIT: Electrical impedance tomography
*P*_aw_: Airway pressure
*P*_es_: Esophageal pressure
*P*_pl_: Pleural pressure
*P*_plat_: Plateau pressure
*P*_L_: Transpulmonary pressure
Clung: Lung compliance
*C*_cw_: Chest-wall compliance
EELV: End-expiratory lung volume
EELI: End-expiratory lung impedance
