# Supplementary material for: External chest-wall compression in prolonged COVID-19 ARDS with low-compliance: a physiological study
Source: Ann Intensive Care. 2022 Apr 12;12:35. doi: 10.1186/s13613-022-01008-6 (PMC9003155; doi:10.1186/s13613-022-01008-6)
Supplement: Supplementary file 1 — Additional file 1. Contains legends for additional figures and additional tables. [file 13613_2022_1008_MOESM1_ESM.docx]

**External Chest-Wall Compression in COVID-19 ARDS with Low-Compliance: a Physiological Study.**

L. Bastia^1^, E. Rezoagli^2,3^, M. Guarnieri^4^, D. Engelberts^5^, C. Forlini^4^, F. Marrazzo^4^, S. Spina^4^, G. Bassi^4^, R. Giudici^4^, M. Post^5^, G. Bellani^2,3^, R. Fumagalli^2,4^, L. J. Brochard^6^*, T. Langer^2,4^*

**ONLINE SUPPLEMENT**

**FIGURE LEGENDS**

**Figure E1. Study protocol.** Protocol made of 4 steps (4 squares in the picture), PEEP was kept constant for the first 3 steps and then reduced for the last step. ECC: external chest-wall compression. + = brief ECC during an end-expiratory hold maneuver, * = static respiratory mechanics measurements, § = arterial and venous blood gas samples collection.

**Figure E2. Brief chest compression during baseline.** In order to detect the pressure applied to respiratory system from a 5 lt saline bag above the thorax we performed this brief maneuver during an expiratory pause. 1) Pressure wave-form of a volume-controlled ventilation modality; 2) expiratory hold starts; 3) application of 5 lt saline bag; 4) increased airway pressure caused by the 5 lt saline bag detected by the ventilator; 5) 5 lt saline bag removed. Paw = airway pressure.

**Figure E3. Delta end-expiratory transpulmonary pressure (P_Lexp_) and delta end-inspiratory transpulmonary pressure (P_Linsp_) compared to baseline.** N = 9, Panel A shows the change in P_Lexp_ for each time-point compared to baseline (gray dashed line), no significant differences were found although the ECC led to a decrease of P_Lexp_. Panel B shows the change in P_Linsp_ for each time-point compared to baseline (gray dashed line). ECC reduced significantly P_Linsp_, as for driving pressure the ECC effect on P_Linsp_ in influenced by time. ECC: external chest-wall compression. * = P < 0.05 of absolute values of different timepoints versus baseline.

**Figure E4. Tidal Volume (V_t_) Distribution.** N = 11. Panel A, V_t_ distribution expressed in % across steps: ventilation is predominantly non-dependent (white dots) and is fairly stable during the protocol. Panel B, V_t_ distribution expressed in delta % from baseline: ECC led to significant increase in the non-dependent ventilation after 5 minutes and after PEEP reduction. However, this difference compared to baseline is lost at 30 and 60 min of ECC. ECC: external chest-wall compression. * = P < 0.05 of absolute values of different timepoints versus baseline.

**Figure E5. Change in Non-Dependent Tidal Volume (V_t_) produced by ECC VS change in Non-Dependent V_t_ produced by PEEP reduction, expressed in % compared to Baseline.** N = 11. Relationship between non-dependent V_t_ variations obtained after 5 minutes of ECC (5 min ECC non-dependent V_t_ % – baseline non-dependent V_t_%) and non-dependent V_t_ variations obtained after PEEP reduction (PEEP reduction non-dependent V_t_ % – baseline non-dependent V_t_%). A linear relationship in the degree of V_t_ change produced by ECC and PEEP reduction was observed (R = 0.72, R^2^ = 0.51, P = 0.01). Suggesting that ECC and PEEP reduction generates similar redistribution in regional ventilation. ECC: external chest-wall compression.

**Figure E6. Representative behavior of end-expiratory lung impedance (EELI) before and after an ECC.** From to bottom EELI of global lung, region of interest (ROI) 1, ROI 2, ROI 3 and ROI 4. Black dashed line: ECC start, red solid line: EELI baseline, red dashed line: EELI right after ECC. Importantly to underline that 1) EELI is reducing over time globally and 2) EELI is reducing mainly in ROI 2. ECC: external chest-wall compression.

**Figure E7. Delta end-expiratory lung impedance (EELI) during ECC.** N = 11. The sustained ECC led to a decrease of ELLI (a surrogate of end-expiratory lung volume) in the non-dependent lung (white dots) reaching statistical significance after 60 minutes compared to the onset of ECC placement (i.e. 5 minutes). The EELI in the dependent lung (black dots) did not change over time. ECC: external chest-wall compression. * = P < 0.05 of differences between the absolute values of timepoints at 30 and 60 minutes versus 5 minutes.

**Figure E8. Bland-Altmann Plot, difference in driving pressure decrease between ECC and PEEP reduction.** The graph shows a good agreement between the two procedures. Bias = -0.86, upper limit of agreement = 0.40, lower limit of agreement = -2.13. DP: driving pressure, ECC: external chest-wall compression. DP: driving pressure, LOA: limit of agreement.

**Figure E9. Spaghetti plot of Driving Pressure across study steps.** Every line represents a single patient throughout the protocol. ECC: external chest-wall compression.

**Figure E10. Spaghetti plot of Lung compliance (A) and chest-wall compliance (B) across study steps.** Every line represents a single patient throughout the protocol. To note, patients #9 and #11 are not reported because of the absence of esophageal balloon. ECC: external chest-wall compression.

**Figure E11. Spaghetti plot of Dorsal Fraction of Ventilation across study steps.** Every line represents a single patient throughout the protocol. This graph represents the percentage of ventilation received from the dorsal part of the lung (*i.e.*, dependent). Grey dashed line = 50% = equal distribution between dorsal and ventral. 0% means no ventilation in the dorsal lung. ECC: external chest-wall compression.

**Figure E12. Spaghetti plot of Non-dependent (A) and Dependent (B) regional C_rs_ across study steps.** Every line represents a single patient throughout the protocol. ECC: external chest-wall compression. C_rs_: respiratory system compliance.

**Figure E13. Respiratory System Pressure-Volume (PV) curve at baseline and after a quick external chest compression (ECC), possible mechanism.** PV-curve baseline (light blue solid curve) is shifted downward from ECC (light blue dashed line) because of the end-expiratory volume reduction (Y axes). PEEP (black dots) does not change however, for the same amount of tidal volume (VT), plateau pressure (P_plat_) at baseline is higher compared to P_plat_ after ECC (red dots). This change in P_plat_ implies a change in driving pressure (DP) on the X axes with lower DP associated with ECC.

**TABLES**

**Table E1. Esophageal balloon positioning**

|  |  |
| --- | --- |
| Distance from nose to balloon of the esophageal catheter (cm) | 39 ± 3 |
| ΔP_es_/ΔP_aw_ | 1.01 ± 0.15 |

Data are expressed as mean ± standard deviation. ΔPes: variation of esophageal pressure caused by a chest compression performed during an end-expiratory hold. ΔPaw: variation of airway pressure caused by a chest compression performed during an end-expiratory hold.

**Table E2. Respiratory Mechanics Variables Through Steps**

|  | Baseline | ECC 5 min | ECC 30 min | ECC 60 min | ECC Disc | PEEP Reduction |
| --- | --- | --- | --- | --- | --- | --- |
| DP  (cmH_2_O) | 14.2 ± 1.3 | 12.3 ± 1.3*  -11.7%  (-21.6 –  -8.2%) | 12.7 ± 1.4*  -7.7%  (-16.2 –  -3.5%) | 13.3 ± 1.5*  -4.8%  (-12.4 – +2.8%) | 14.9 ± 1.8  +0.7%  (-0.1% – +10.0%) | 13.1 ± 1.3*  -6.2%  (-15.8 – 0.0%) |
| PEEP_t_ | 12.6 ± 2.9 | 12.8 ± 2.9 | 12.6 ± 2.9 | 12.6 ± 3 | 12.6 ± 3 | 9.8 ± 2.9* |
| P_plat_  (cmH_2_O) | 27  (26 – 28.2) | 24.6  (22.8 – 27.7)  -5.7%  (-8.8 – -4.2%) | 25  (22.8 – 28)  -5.0%  (-8.8 –  -0.7%) | 25.7  (23 – 29)  -3.7%  (-7.1 – 2.8%) | 28.4  (26.4 – 29)  +0.7%  (0.0 – +5.9%) | 22.5*  (20 – 24.8)  -13.5%  (-18.5 –  -12.1%) |
| C_rs_  (mL/cmH_2_O) | 25.9 ± 5.9 | 30.2 ± 7.8*  +16.3 ± 11.7% | 29.2 ± 7.7*  +12.2% ± 10.3 | 28.0 ± 7.7  +7.4 ± 11.8% | 24.9 ± 6.3  -4.3 ± 6.8% | 28.3 ± 7.8*  +8.8 ± 12.6% |
| EL_rs_  (cmH_2_O/mL) | 37  [34 – 45] | 31*  [28 – 41] | 33  [29 – 41] | 34  [32 – 44] | 40#  [35 – 45] | 33  [32 – 45] |
| C_cw_  (mL/cmH_2_O) | 152  (120.2 – 305.1) | 172.7  (108.7 – 295.2)  -4.0%  (-9.8% – +7.5%) | 153.8  (109.6 – 348.9)  -7.6%  (-16.2 – +30.6%) | 160  (96.3 – 326.7)  -13.3%  (-24.0 – +10.0%) | 187.5  (151.5 – 312.8)  +11.1%  (-6.0 – +34.5%) | 327.3  (151.5 – 401.1)  +18.2%  (+4.5 – +102.2%) |
| EL_cw_  (cmH_2_O/mL) | 6 ± 2 | 6 ± 3 | 6 ± 3 | 6 ± 3 | 5 ± 1 | 4 ± 2 |
| C_lung_  (mL/cmH_2_O) | 28.7 ± 6.1 | 35.5 ± 9.3*  +23.4 ± 14.9% | 33.3 ± 8*  +15.9 ± 11.3% | 32.1 ± 7.9  +12.0 ± 16.2% | 26.5 ± 6.3§  -7.5 ± 9.2% | 30.3 ± 6.3  +6.3 ± 10.6% |
| EL_lung_  (cmH_2_O/mL) | 33  [29 – 42] | 27*  [22 – 37] | 28*  [24 – 38] | 30  [25 – 42] | 39#  [32 – 44] | 31  [29 – 39] |
| MAP  (cmH_2_O) | 17  (15 – 20) | 17  (15 – 20) | 17  (15 – 20) | 17  (15 – 20) | 17  (15 – 21) | 14*  (12 – 17) |
| Non-Dependent Regional C_rs_  (mL/cmH_2_O) | 16.4 ± 5.6 | 19.8 ± 6.6*  +16.9%  (+14.3 – +33.7%) | 18.9 ± 6.7*  +14.1%  (+9.4 – +19.3%) | 17.9 ± 6.5*  +11.8%  (+1.0 – +18.8%) | 15.7 ± 5.1  -2.7%  (-7.4 –  -0.01%) | 18.4 ± 6.1*  +15.4%  (+2.8 – +23.6%) |
| Dependent  Regional C_rs_  (mL/cmH_2_O) | 9.4 ± 2.2 | 10.3 ± 2.8  +5.8 ± 9.5% | 10.2 ± 2.7  +3.9 ± 10.6% | 9.9 ± 2.5  +2.2 ± 8.6% | 9 ± 2.5  -5.1 ± 8.9% | 9.7 ± 3.1  +2.5 ± 15.8% |
| End-Expiratory P_L_  (cmH_2_O) | -1.7  (-4.8 – 0.25) | -3.1  (-5.6 – -1.5)  -135.3%  (-313.3 –  -21.6%) | -3.5  (-5.7 – -1.4)  -109.1%  (-344.6 –  -35.9%) | -3  (-5.6 – -0.5)  -72.7%  (-148.5 –  -38.4%) | -0.4  (-2 – 1.7)  +36.4%  (+2.3 – +124.5%) | -2.5  (-4.4 – 0.7)  -55.2%  (-182.6 –  +8.5%) |
| End-Inspiratory P_L_  (cmH_2_O) | 11.7 ± 4.3 | 7.7 ± 5*  -38.9 ± 18.4% | 8.1 ± 5.2*  -34.9 ± 17.7% | 9.1 ± 5.1*  -25.3 ± 19.1% | 13.9 ± 4.3  +21.4 ± 16.2% | 10 ± 4.4  -15.3 ± 18.0% |

Data are expressed as median and interquartile range or mean ± standard deviation as appropriate. Percentages are expressed compared to baseline. ECC: external chest-wall compression, DP: driving pressure, P_plat_: plateau pressure, C_rs_: respiratory system compliance, EL_rs_: respiratory system elastance, C_cw_: chest-wall compliance, EL_cw_: chest-wall elastance, C_lung_: lung compliance, EL_lung_: lung elastance, MAP: mean airway pressure, P_L_: transpulmonary pressure. *: P < 0.05 compared to baseline. § = P < 0.05 compared to ECC at 5, 30 and 60 minutes. # = P < 0.05 compared to ECC at 30 and 60 minutes.

**Table E3. Gas Exchange over time**

|  | Baseline | ECC 60 min | ECC Discontinuation | PEEP Reduction | P-value |
| --- | --- | --- | --- | --- | --- |
| P_a_O_2_ (mmHg) | 88.2  (77.1 – 127.6) | 71.6  (66.2 – 85.4)  -7.3%  (-20.1 – +1.1%) | 84.9  (68.1 – 134.8)  -0.4%  (-8.1 – +4.3%) | 85.7  (66.8 – 129.5)  -0.6%  (-14.7 – +13.2%) | 0.20 |
| P_a_CO_2_ (mmHg) | 55.9 ± 6.6 | 53.5 ± 6.9  -6.0%  (-8.5 – +0.4%) | 53.1 ± 6.7  -5.1%  (-10.6 – +1.1%) | 52.2 ±8.2  -3.9%  (-10.0 – +0.6%) | 0.06 |
| pH | 7.371 ± 0.03 | 7.392 ± 0.03 | 7.386 ± 0.03 | 7.397 ± 0.03***** | 0.01 |
| P_a_O_2_/F_i_O_2_ | 163  (109 – 220) | 136  (109 – 200) | 150  (113 – 202) | 162  (110 – 227) | 0.20 |
| F_i_O_2_ (%) | 60  (50 - 65) | 60  (50 - 65) | 60  (50 - 65) | 60  (50 - 65) | 1.00 |
| End-tidal CO_2_ (mmHg) | 42  (39 – 50) | 42  (37 – 45) | 42  (39- 49) | 41  (37 – 46) | 0.08 |
| Dead space (%) | 18.6  (12.6 – 27.1) | 20  (14.7 – 26.7) | 14.7  (7.5 – 24) | 18  (11.4 – 26.9) | 0.24 |
| Ventilatory Ratio | 2.1 ± 0.4 | 2.07 ± 0.5 | 2.08 ± 0.4 | 2.02 ± 0.5 | 0.21 |
| Shunt fraction (%) | 15.5  (7.1 – 27.3) | 19.0  (7.0 – 25.0) | 18.9  (7.1 – 27.0) | 15.0  (9.6 – 25.6) | 0.69 |
| P_A_O_2_-P_a_O_2_ | 254.4  (162.0 – 327.2) | 266.4*****  (164.1 – 328.7) | 253.7  (163.1 – 321.4) | 245.1  (149.6 – 327.8) | 0.02 |
| P_A_O_2_/P_a_O_2_ | 3.48  (2.60 – 5.55) | 4.21  (2.90 – 5.60) | 3.81  (2.73 – 5.29) | 3.48  (2.28 – 5.47) | 0.09 |

Data are expressed as median and interquartile range or mean ± standard deviation as appropriate. Percentages are expressed compared to baseline. ECC: external chest-wall compression. *: P < 0.05 compared to baseline.

**Table E4. Hemodynamics over time**

|  | Baseline | ECC 5 min | ECC 30 min | ECC 60 min | ECC Discon | PEEP Reduction | P-value |
| --- | --- | --- | --- | --- | --- | --- | --- |
| HR  (bpm) | 93 ± 14 | 92 ± 13 | 91 ± 12 | 90 ± 11 | 93 ± 9 | 91 ± 14 | 0.50 |
| SAP (mmHg) | 126 ± 16 | 132 ± 19 | 135 ± 18 | 128 ± 12 | 128 ± 15 | 130 ± 17 | 0.30 |
| MAP (mmHg) | 79 ± 17 | 80 ± 13 | 84 ± 15 | 77 ± 12 | 77 ± 12 | 76 ± 16 | 0.10 |
| DAP (mmHg) | 67 ± 11 | 68 ± 5 | 70 ± 10 | 64 ± 12 | 66 ± 9 | 64 ± 11 | 0.40 |
| CVP (mmHg) | 9 ± 3 | 11 ± 3 | 10 ± 3 | 9 ± 3 | 9 ± 4 | 10 ± 3 | 0.06 |

Data are expressed as mean ± standard deviation as appropriate. ECC: external chest-wall compression, HR: heart rate, SAP: systolic arterial pressure, MAP: mean arterial pressure, DAP: diastolic arterial pressure, CVP: central venous pressure.
