# Supplementary figures and images for: External chest-wall compression in prolonged COVID-19 ARDS with low-compliance: a physiological study
Source: Ann Intensive Care. 2022 Apr 12;12:35. doi: 10.1186/s13613-022-01008-6 (PMC9003155; doi:10.1186/s13613-022-01008-6)

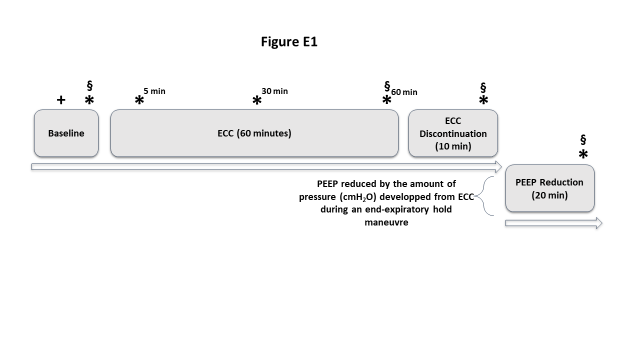


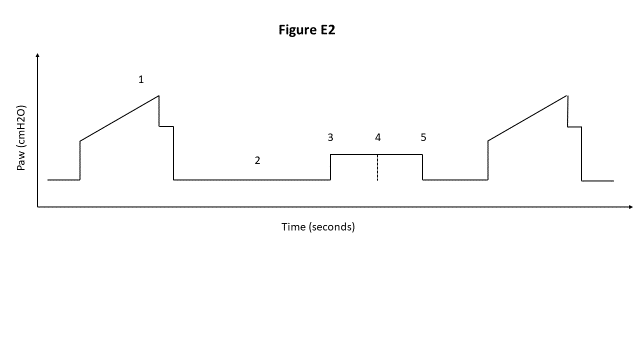


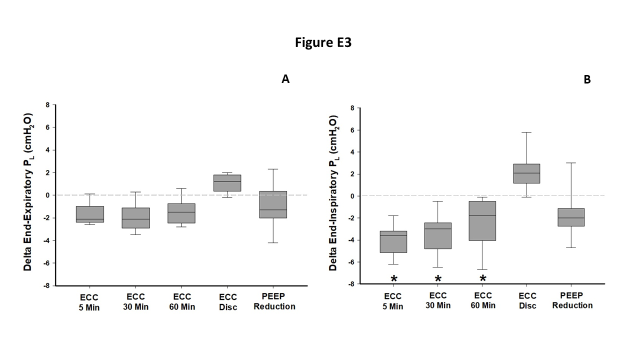


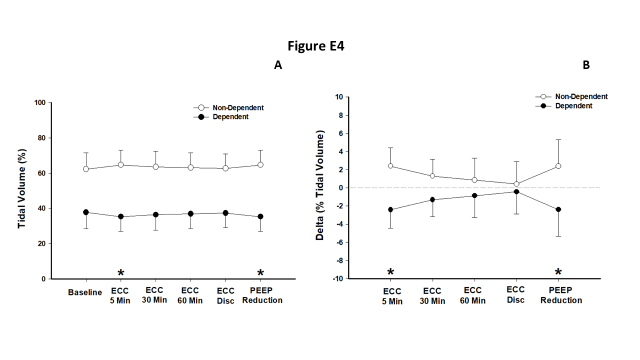


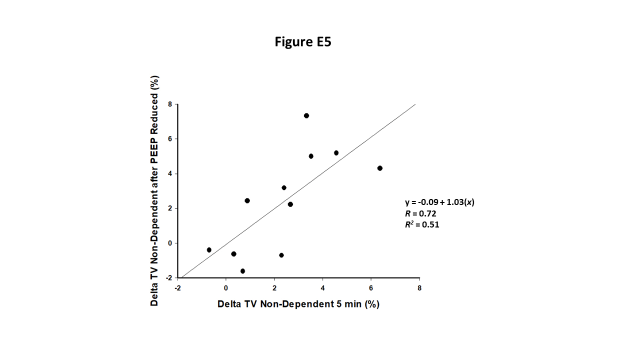


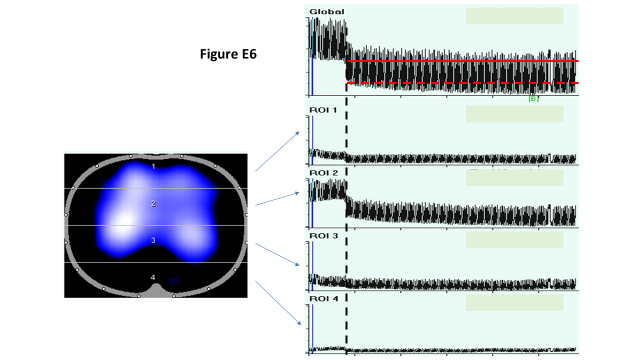


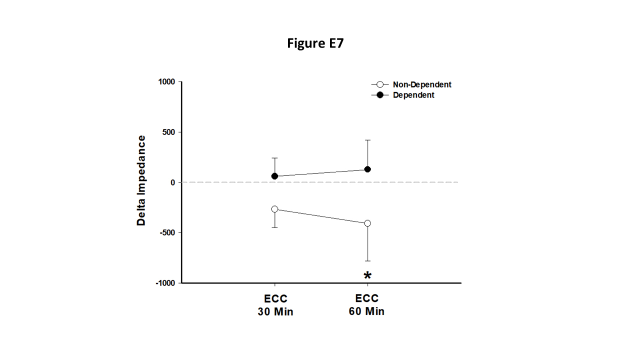


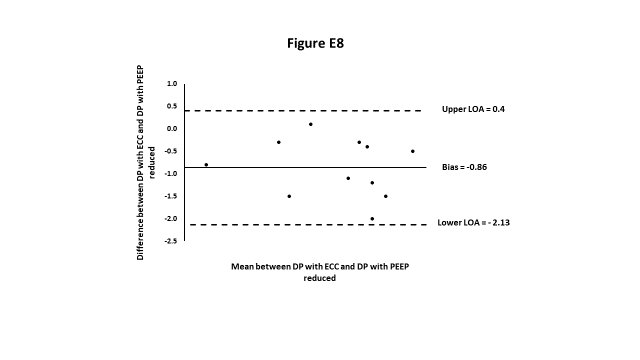


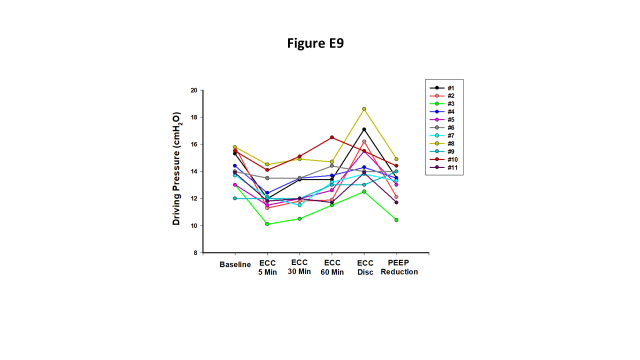


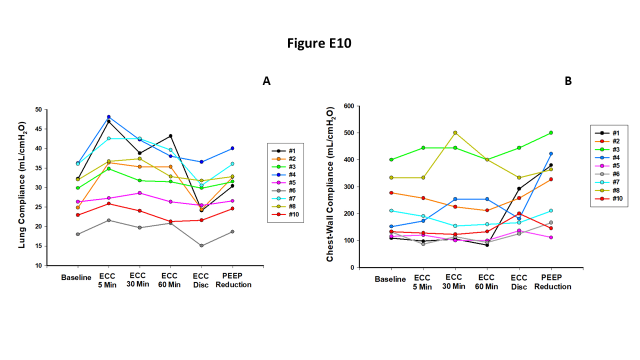


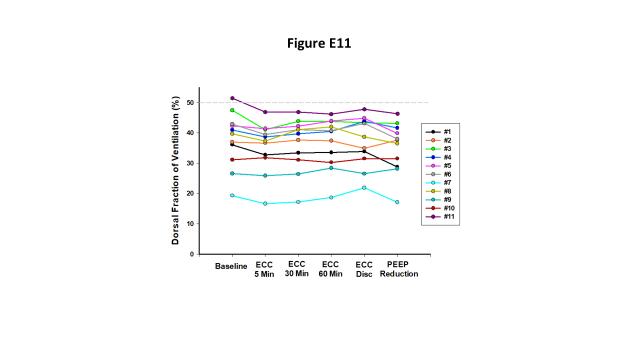


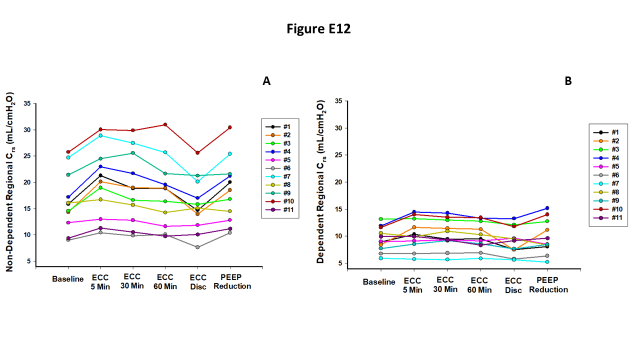


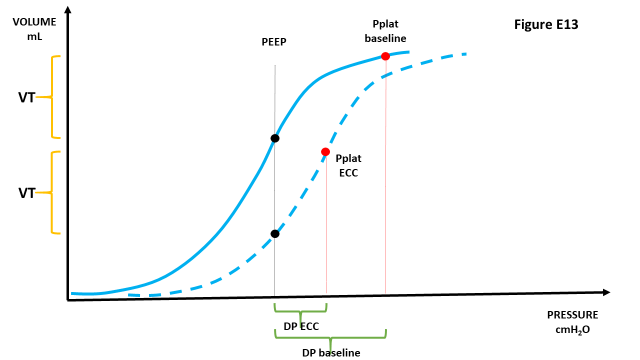

Supplement: Supplementary file 2 — Additional file 2. Contains additional figures. [file 13613_2022_1008_MOESM2_ESM.docx]
